# The Effects of Case-Based Team Learning on Students’ Learning, Self Regulation and Self Direction

**DOI:** 10.5539/gjhs.v7n4p295

**Published:** 2015-01-25

**Authors:** Rita Rezaee, Leili Mosalanejad

**Affiliations:** 1 Quality Improvement in Clinical Education Research Center, Shiraz University Of Medical Sciences, Shiraz, Iran; 2 Mental Health Department, Jahrom University of Medical Sciences, Shiraz University of Medical Sciences, Iran

**Keywords:** Case-based learning, Team-based learning, Self-regulated learning, Self-directed learning, long life learning, medical education

## Abstract

**Introduction:**

The application of the best approaches to teach adults in medical education is important in the process of training learners to become and remain effective health care providers.

This research aims at designing and integrating two approaches, namely team teaching and case study and tries to examine the consequences of these approaches on learning, self regulation and self direction of nursing students.

**Material & Methods:**

This is aquasi experimental study of 40 students who were taking a course on mental health. The lessons were designed by using two educational techniques: short case based study and team based learning. Data gathering was based on two valid and reliablequestionnaires: Self-Directed Readiness Scale (SDLRS) and the self-regulating questionnaire. Open ended questions were also designed for the evaluation of students’with points of view on educational methods.

**Results:**

The Results showed an increase in the students’ self directed learning based on their performance on the post-test. The results showed that the students’ self-directed learning increased after the intervention. The mean difference before and after intervention self management was statistically significant (p=0.0001). Also, self-regulated learning increased with the mean difference after intervention (p=0.001). Other results suggested that case based team learning can have significant effects on increasing students’ learning (p=0.003).

**Conclusion:**

This article may be of value to medical educators who wish to replace traditional learning with informal learning (student-centered-active learning), so as to enhance not only the students’ ’knowledge, but also the advancement of long- life learning skills.

## 1. Introduction

Providing healthservices is only possible when the graduates are ableto adapt themselves to the continuous development of medical knowledge, clinical environmental complexityandrapid changesin technology (Distler, 2007; Worrell & Profetto-McGrath, 2007). It is also essential for learners to possess the necessary abilities to encounter different situations and solve their patients’ problems, particularly in vital situations (Ozturk, Muslu & Dicle, 2008).

It is also worth noting that in the field of education, strengthening and developing such skills, problem solving, critical thinking, interpersonal skills, and creativity, are highly important; it is also necessary to design such an educational environment as to provide the possibility of linking theoretical training with real- life situations (Vahidi, Azemian, & Vali Zadeh, 2004). Therefore, meting the need to create an appropriate training environment, applying modern methods of skill training, and improving the current methods are recommended (Abraham, 1995). Increasing needs in science and technology have necessitated the application of modern teaching and training methods, among them active student-centered methods (Hadges & Videto, 2005).

Self-regulatory skills and self-direction in learning are two important skills which are essential to developing skills in teaching, along with problem solving skills and the choice of teaching methods.

**Figure 1:**
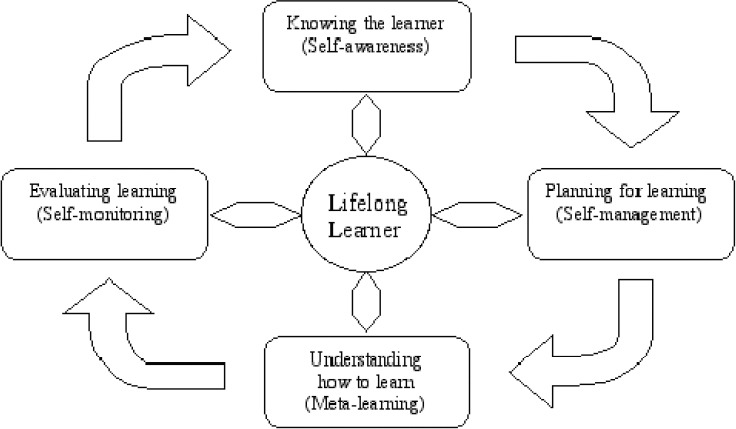
Characteristics of lifelong learners

The concept of “lifelong learning” is considered as a key to success in the 21st century. To achieve that, it is necessary that the following guidelines be implemented (Figure 1)

Over the next decade, the cognitive limitations of the traditional models, where physicians are expected to learn, retain, and keep up with an ever-expanding body of medical knowledge, will become more acute. New models for learning and sharing will be needed (Braddock, Eckstrom, & Haidet, 2004; Kaufman & Mann, 2010; McGowan et al., 2012; Yousefy & Gordanshekan, 2010).

**Figure 2:**
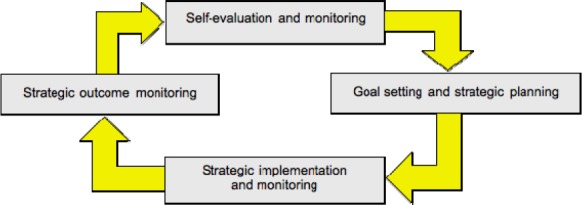
The process of Self regulated learning

Self-regulation is a comprehensive construct that involves complex interactions among cognitive, metacognitive and motivational strategies (Boekaerts, Pintrich, & Zeidner, 2000; Butler, 2011) (Figure 2).

**General model**

General models of regulation express that although learners are simultaneously directed and limited by their goals and contextual characteristics, they set goals for their learning and try to regulate, monitor and control their motivation, cognition, and behavior.

A second assumption is that learners can potentially monitor, control, and regulate certain aspects of their own cognition, motivation, behavior and some features of their environment as well.

A third assumption is that there is some type of criterion or standard against which comparisons are made to assess if the process should continue as is or if some types of change are required.

Therefore, self regulatory activities are mediators between personal and contextual features on one hand and actual achievement or performance on the other (Butler, 2011; Zimmerman, Bandura, & Martinez-Pons, 1992; Boekaerts et al., 2000).

Due to the advantages of self-directing learning, training and organizational environments emphasize its importance and its value as a necessary skill for training and work in the twentieth century (Avalos, 2011; Duță et al., 2013; Hiemestra, 1994; Braddock et al., 2004).

Different scholars have presented different definitions of SDL. According to Candy (1991), as an umbrella concept, SDL encompasses four dimensions: ‘self-direction as a personal attribute (personal autonomy); self-direction as the willingness and capacity to conduct one’s own education (self-management); self-direction as a mode of organizing instruction in formal settings (learner-control); and self-direction as the individual, non-institutional pursuit of learning opportunities in anatural societal setting.

Brockett and Hiemstra (1991) provided a rationale for two primary orientations: process and goal. Garrison (1997) maintained that SDL is accomplished through thethree dimensions of self-management, self-monitoring, and motivation, which interact with each other. In Pintrich’s model, self-regulated learning is defined as a cognitive and metacognitive strategy which incorporates resource management (Pintrich & Zusho, 2002; Abraham, 1995; Arbarca, Mana & Gil, 2010; Brockett & Hiemstra, 1991; Butler, 2011) (Table 1).

**Table 1:** Perspectives on Self-Directed Learning

Perspective	Description	Model		

		Canday (1991)	Brockett & Hiemstrs (1991)	Garrison (1997)
Personal Attribute	Moral, emotional And intellectual management	personal autonomy self- management	Goal orientation (personal attribute)	self-management (Use of resoures) Motivation
Process	Learner autonomy Over instruction	Learner control Autodidaxy	process orientation learner control	Self manitoring
Contex	Environment where learning takes place	Self direction is context bound	Social context role of institutions and policies	

The role of the educator is to move from the role of a wise person in the learning process to the creator of a self-directing learning environment (Fisher, King, & Tague, 2001).

In addition, various reviews ofthe progressive medical education program indicate that a majority of these programs neither change the students’ behavior, nor affect patients’ recovery results, and in simple words, are not based on the needs of learners (Davis et al., 1999; Mazmanian & Davis, 2002).

The American Board of Internal Medicine (ABIM) suggests that it is necessary for a teacher to be a lifelong learner and engage in a periodic self-assessment process; continuous learning should be a requirement forthe maintenance of one’s certificate..

Due to the difficulty of training learners in mental health and psychiatric courses, and considering the fact that that many of the signs and symptoms of these patients, as well as learning how to communicate with them, are difficult parts in these fields, methods that will attract the full attention of students and provide a greater possibility of understanding for them are more useful. In addition, regarding the fact that in the area of combined learning methods, few studies, if any, have been conducted, this research aimed to design and integrate models of the two approaches of team teaching and case study to investigate the effects of this method on learning, self-regulation, and self-direction.

## 2. Material and Methods

### 2.1 Participants

This quasi-experimental (quasi experimental with a non-equivalent control group design)

wasconducted on 40 nursing students who were taking a psychiatry course. It was a two-credit course taught as a specialized course in the undergraduate curriculum. In this method, which was implemented during one academic semester, purposive sampling was used.

### 2.2 Study Design

The purpose of this study was to test the effects of explicit teaching of psychiatry through case-based team learning on teaching and learning, and see if it would produce an improvement in the performance, self regulation and self direction of nursing students.

The teaching process used here wasextracted from two teaching techniques based on real cases (case studies) that were adjusted, according to the specialized resources and teachers’ experiences, as a problem-based mixed method of teaching.

Then, in addition to examining the issues proposed individually and as a team, the students investigate and discuss the issues. At first, the instructor teaches the diseases and after completing the basic discussions in each course, he/she suggests studies concerning communication therapy-how to communicate with patients and their families. Then, the therapeutic challenges are presented as a case study or a short clinical problem. Then, the students have one week to find a logical answer to the challenge. The questions do not have a definitive answer, but are related to divergent and productive thinking of the students to explore different aspects of each issue. The references should be specified in the first session from updated textbooks and Internet resources. All of the students were trained during makeup sessions in using digital libraries, classification and application. Students used these sources for searching and finding responses to the problems determined as a case based or problem based issue in the course. This part of the study was a type of web quest in the students’ learning process. Case based, team based, and individual and group web quest is a blended teaching- learning process that promotes students’ learning.

After a week of self-study and teamwork, the students reviewed the aspects of the issue at the beginning of the class and then made a conclusion; subsequently, the instructor dealt with the most important points. 4 groups, each consisting of 10 students, were chosen in such a way that strong and weak students were equally distributed based on their previous grades and. To increase their ability, each group received a different, but equally tough, issue.


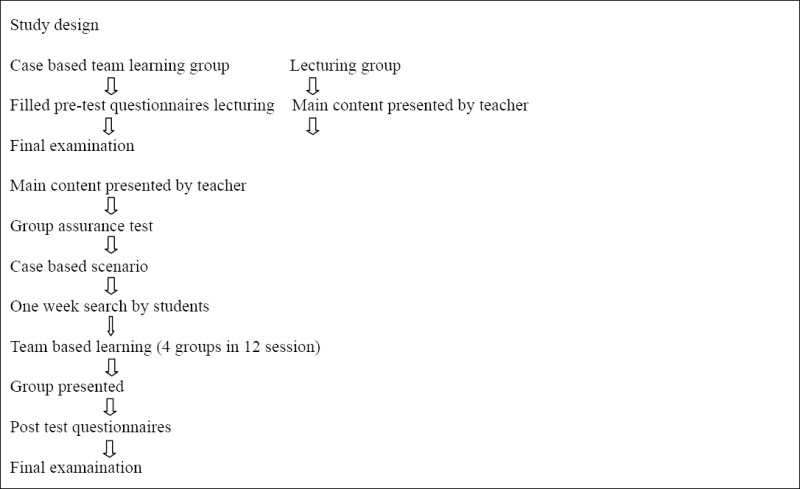


### 2.3 Study Evaluation

In order to promote the students’ learning and to ensure that they were prepared to enter the new phase (individual and group assurance test), the students were asked to answer 4 multiple-choice questions individually or in groups in the last 5 minutes of every session. The results of the questions were reviewed and the students were given feedback. The entire procedure took around 2 hours. Attempt was also made to devote more time to the second session in the morning. The Students ’ learning was evaluated by their final scores and in comparison with their predecessors who were also given the same questions and the same content.

### 2.4 Data Gathering

In order to determine the individuals’ readiness to learn self-regulated and self-directed learning, a standard questionnaire was used. Self-Directed Readiness Scale (SDLRS) is a self report questionnaire with 58 questions in 5- point Likert-type items (ranging from hardly ever to always) which includes three areas: self-management, willingness to learn, and self-control. The internal consistency and test-retest reliability were calculated to be 0.95 and 0.68, respectively. Scores were calculated out of 100 for each area. Scores less than 33.3 were considered as low, between 33.3 to 66.7 as average, and more than 66.7 as high cronbach’s alpha coefficients for the subscales of self-management, willingness to learn, and self control were reported to be respectively 0.81, 0.78, and 0.84 (Guglielmino, 1997).

The second questionnaire which was related to self-regulation, contains 14 questions has been designed by Buford et al (1995), and was normalized in the Iranian society. The overall reliability was 0.71 based on cronbachs’ alpha coefficient. The reliability of the Subscales of cognitive and meta cognitive strategies were reported to be 0.7 and 0.68, respectively. Construct validity was satisfactory. In this test, five options were considered for each question ranging from “completely agree, agree, no idea, disagree, and completely disagree” and were rated from 1 to5, respectively (Fisher, King, & Ague, 2001; Nadi & Sajadian, 2011; Smedley, 2007; Zimmerman et al., 1992; Zimmerman & Schunk, 2013).

Satisfaction with and the effects of the teaching methods were examined through open-ended questions at the end of the last session and were qualitatively analyzed.

Results: Out of the 41 students who participated in the project, 26 were female, and most of them were in the same age range (20-25 years).

Data distribution showed an increase in the students’ self-directed learning, based on their performance on the post test (Table 2).

**Table 2:** Distribution of self directed learning in groups before and after intervention

Variable		Before		After		

		Frequency	%		Frequency	%
Self-directed learning	Very low	-	-	Very low	-	-
	Low	-	-	Low	-	-
	Moderate	3	7.5%	Moderate	-	-
	High	29	72.5%	high	31	77.5%
	Very high	8	20.%	Very high	9	22.5%

The results showed that, although self-directed learning had increased after the intervention, there were significant differences among the students’ levels of self management after intervention (p=0.0001),Also, self-regulated learning increased with the mean difference(p= 0.001) (Table 3).

**Table 3:** The mean difference of students’ learning indices before and after intervention

variable		Mean (SD)	
Self-directed learning	before	Self regulation	58.72(5.02)
		Desire for learning	55.26(5.11)
		Self management	46.6(4.37)
		Total	68.47(6.41)

	after	Self regulation	59.06(4.89)
		Desire for learning	55.44(4.61)
		Self management	50. 6(4.46)*
		Total	69.90 (5.36)

Self-regulated learning	before	48(4.89)	

	after	52.65 (5.21)*	

P from paired t- test;

* p is significant (p<0.05);

Self regulation (p=0.78), Desire for learning (p=0.87), Self management (p=0.0001), Self regulated learning (p=0.001).

Other results suggested that the case based team approachcan have significant effects on increasing students’ learning (Table 4).

**Figure 3:**
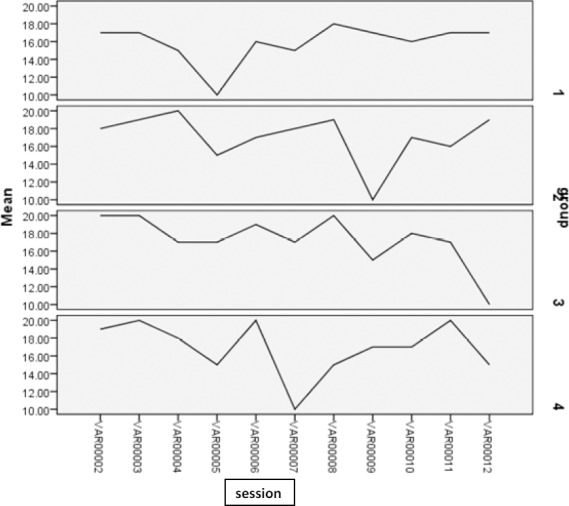
Mean score of self assurance test in group session

**Figure 4:**
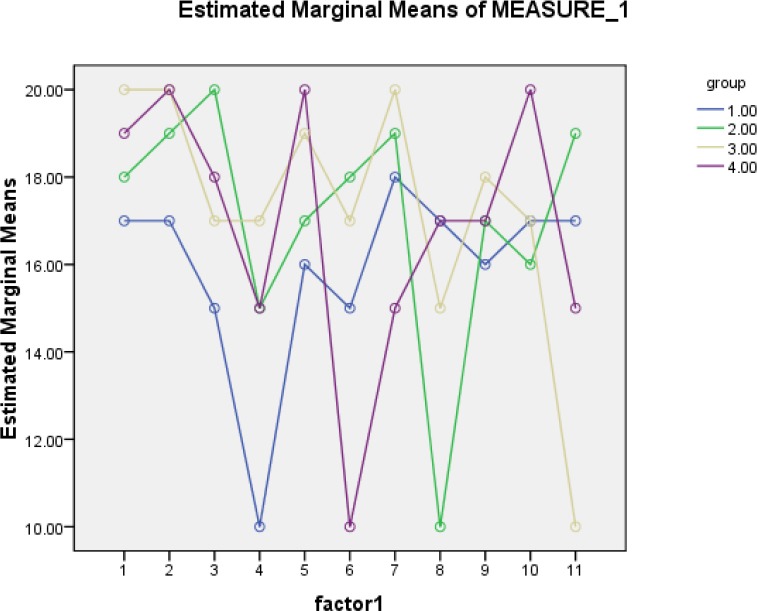
Group assurance test in all groups

In another part of the study, the readiness assurance test was applied in 12 sessions. The mean score of group assurance test is shown below. This part showed that, regarding the levelofdifficulty in understanding contents, the groups had different approaches to responding the questions. All the groups tried to do their best during team work. Some groups achieved better results with regard to multiple choice questions, but each group tried to out do the other groups in this contest. Increasing and decreasing scores showed the differences among the teams’ activities as groups, as well as differences in understanding contents. (Figures 3 and 4).

**Table 4:** The mean difference of the scores of the students in based team learning group and traditional group

Teaching status	Mean (SD)	T
Intervention group(n=40)	14.87 (1.89)	18.3*
Traditional (n=41)	13.24 (2.01)	

* P= 0.003.

In another analysis, open-ended questions were used to examine the students’ viewpointson this approach. The results of the analysisled to identifying the impact of these methods on students’ learning. Studentsstated thatthis method madelearningfun and enjoyable (87%), providedthe opportunityfor deeperlearning (45%) and changed their roles from sheer listeners to active learners (48%). Increasing the students’ participation in their own learning (23%), attempt for learning (38%), searching for resources andpreparation forclass (13%), and not being traditionaland stereotyped (11%) were among other characteristics referred to by the students.

## 4. Discussion

The results of the study revealed the contribution ofproblem-orientedlearning method through applying case studies and group discussions to learning. Also, the results showed that self-directed learning had increased with the mean difference.

Self-regulation is associated with academic achievement (Alibakhshi & Zarea, 2010; Kaufman & Mann, 2010; Nota, Soresi, & Zimmerman, 2004; Pesut, 2004; Sobhaninzhad & Abedi, 2006; Zimmerman & Schunk, 2013). Teaching self-directed learning can enhance educational achievement and boost learning motivation (Loyens, Magda, & Rikers, 2008; Mohsenpoor, Hejazi, & Kiamanesh, 2007). The above-mentioned facts confirm the effectiveness of this educational method and its contributions to educational improvement.

The results demonstrated that team learning improves students’ final scores and their understanding, increases the possibility of content retention, encourages critical thinking, improves their attitudesto thecourse, and enhances students’ interaction with learning, which plays an important role in learning and achieving higher scores (Michael, McInerney, & Dee, 2003).

An Investigation of the effects of this approach on 178 medical students indicated that this method considerably improves students’ performance, which is achieved through students’ mastery ofthe contents taught.

In another study, the three universities of California, Los Angeles (UCLA), and California (Davis) were compared using two problem-based methods and a case study from the viewpoints of teachers and students in three consecutive years. In this study, the case study approach was preferred by 89% of the students and 84% of the professors. The participants also preferred case study because it involved less contact and less work, and provided better opportunities for the application of clinical skills (Srinivasan et al., 2007).

The results of the present study indicated the effectiveness of this method on increasing students’ self-directed learning. the results of many studiesare alsoconsistent with and confirm the above results. Application of problem-oriented methods makes a great contribution to promoting self-directed learning (Loyens, Magda, & Rikers, 2008).

The study results showed that problem-based learning has noticeable educational outcomes, including students’ satisfaction and enjoyment, deeper learning, student-centeredness, self-learning, searching for resources and preparation for class, and not being traditional and stereotypical. The results of some other studies also confirm students’ preferencefor these methods.

Others have referred to five important factors in team learning, professional people, resources and facilities, time, and features of the curriculum, scoring them 60%, 38%, 37%, 36%, respectively (Thompson et al., 2007).

In addition, problem-based educational interventions can provide conditions and educational context to foster self-directed learning. research shows that it is necessary to review the current teaching methods and educational ethics and make some changes in the compiled curricula to provide an appropriate context for the development of self-directed learning (Loyens, Magda, & Rikers, 2008; Premkumar et al., 2013).

Components of PBL are often included in SDL. This usually occurs when the teacher considers her/himself as afacilitator in the process of learning, rather than the content source, and tries to improve SDL skills and related behaviors; also, learners should learn to set their own goals, identify their learning resources, and perform self-assessment. This is not the case, however, with PBL where the course organizers or the teachers may even state the purposesor include didactics (Kanter, 1998; Towle & Cottrell, 1996).

The teacher plays an important role as learning facilitator, and the student’s role is characterized by such qualities as readiness for learning, learning preferences and learning style, alongside features such as self-confidence, independence, and motivation.

The method of case- based team learning increased the students’ self management. Other studies confirm this result and express that urban students have higher self-directed learning than rural students. Also, learners who have better plans for their learning and have a more efficient self-assessment program enjoy higher self-directed learning (Fasce et al., 2013).

Applying problem-based methods works best forteaching adults and has a significant impact on the formation of self-directed learning and lifelong learning (Lee, Mann, & Frank, 2010; Levett-Jones, 2005; Tagawa, 2008).

Applying methods that put students in real learning environments and provide the context for self-reflection in team scan be effective in promoting self-directed learning. This finding is consistent with the results of the present study (Murad, Coto-Yglesias, Varkey, Prokop, & Murad, 2010).

Also, some studies emphasize the positive effects of high individual skills in searching for learning resources and promoting one’s learning skills and knowledge on self-directed learning.

These studies confirm our results regarding the effects of problem based education on students’ learning skills.

Other results of the study indicated the impact of the problem-based team learning method on self-directed learning. The mean of self-directed learning score improved considerably after intervention.

The evidence indicated that encouraging metacognition or self-reflection and applying methods which are effective in developing critical thinking can result in better self-regulated learning (Kuiper, 2002; Murad et al., 2010).

Teaching and learning clinical reasoning in clinical environments is one of the methods of self-directed learning and development in medical sciences, and puts students in real conditions by case teaching methods and facilitates reasoning both individually and as a team. Self-directed learning increases the possibility to develop the important skills of cognition and metacognition, as well as evaluation and self-reflection, in clinical reasoning in clinical wards (Kuiper, 2002).

Another study has shown that self-directed learning skill scan be enhanced through the three factors of motivation, independence, and control, which, in turn, can be developed by problem-based teaching methods. Teaching skills by this method in early years prepares learners for self-directed learning in coming years and will have the maximum efficiency in transition from the early stages to the clinical stages (Mohsenpoor et al., 2007).

## 5. Limitations

The quasi-experimental nature of the study and limited number of samples were the limitations of the study. It is necessary to carry out the research with larger groups to confirm the results. Also, the acceptance of traditional methods as the logical approach makes teaching groups and performing group training difficult and may also affect the results.

## 6. Conclusion

Given the positive effects of problem-based methods on learning and the role self-directionas a significant component of lifelong learning in students of medical sciences, it is necessary to develop and implement student-centered methods based on learning-oriented theories in universities and provide opportunities for promoting the learning of nursing students as providers of health.
